# Supplementary material for: Interaction of a Polyarginine Peptide with Membranes of Different Mechanical Properties
Source: Biomolecules. 2019 Oct 18;9(10):625. doi: 10.3390/biom9100625 (PMC6843195; doi:10.3390/biom9100625)

Supporting Information for:

Interaction of a polyarginine peptide with membranes of different mechanical properties .

Matías A. Crosio[1,2], Matías Via[3], Candelaria I. Camara[4,5], Agustin Mangiarotti[1,2,6], Mario G. Del Popolo[3] and Natalia Wilke[1,2,*]

[1] Universidad Nacional de Córdoba, Facultad de Ciencias Químicas, Departamento de Química Biológica Ranwel Caputto, Córdoba, Argentina.

[2] CONICET, Universidad Nacional de Córdoba, Centro de Investigaciones en Química Biológica de Córdoba (CIQUIBIC), Córdoba, Argentina.

[3] ICB-CONICET & Facultad de Ciencias Exactas y Naturales, Universidad Nacional de Cuyo, Mendoza, Argentina

[4] Universidad Nacional de Cordoba, Facultad de Ciencias Químicas, Departamento de Fisicoquímica, Córdoba, Argentina

[5] Consejo Nacional de Investigaciones Científicas y Tecnicas, CONICET, Instituto de Investigaciones en Fisicoquímica de Córdoba, INFIQC, Córdoba, Argentina

[6] Current affiliation: Instituto de Investigación Médica Mercedes y Martín Ferreyra (INIMEC-CONICET-Universidad Nacional de Córdoba), Córdoba, Argentina.

* Correspondence: natalia.wilke@unc.edu.ar

Figure S1: Structures of the used lipids
Figure S2: Analysis of the fluorescence of 5-FAM-KR$_9$C on the GUV´s rim
Figure S3: Thermal fluctuation of DOPG/DOPC GUVs
Figure S4: Test for BLM formation
Figure S5: Representative conductivity experiments
Figure S6: Compression isotherms and calculation of ΔA upon peptide incorporation
Figure S7: Size distribution of LUVs in the presence of KR$_9$C
Figure S8: Kinetic of peptide accumulation at the membrane

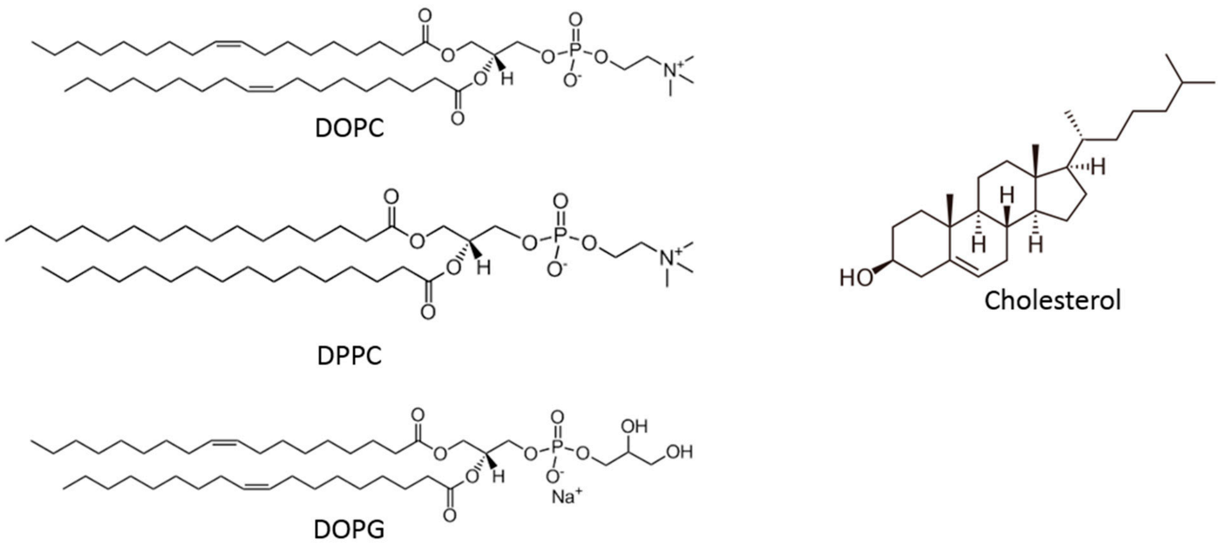

Figure S1: Structures of the used lipids

Figure S2: Analysis of the fluorescence of 5-FAM-KR$_9$C on the GUV´s rim

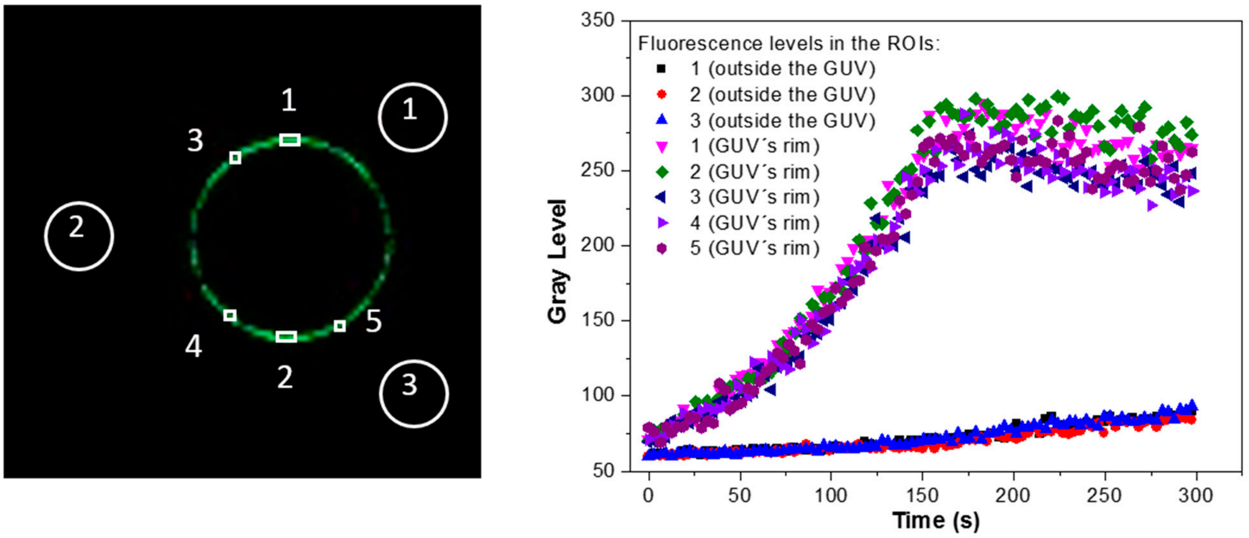

The image shows an example of the ROIs selected for determining the fluorescence outside a GUV and at the GUV´s rim. In the plot, the gray level at each ROI is depicted as a function of time, starting from the time in which the fluorescence increased. The GUV is composed of DOPC/CHOL/DOPG, and 2.5 µM peptide was added.

Figure S3: Thermal fluctuation of DOPG/DOPC GUVs

AR values were determined in a 160 s-long video and the average value along with the related standard deviation (SDAR) was determined. The figures show the values for SDAR at each peptide concentration of a representative experiment, and the corresponding histogram. The black line shows the value of P95 for the control experiment (without peptide).

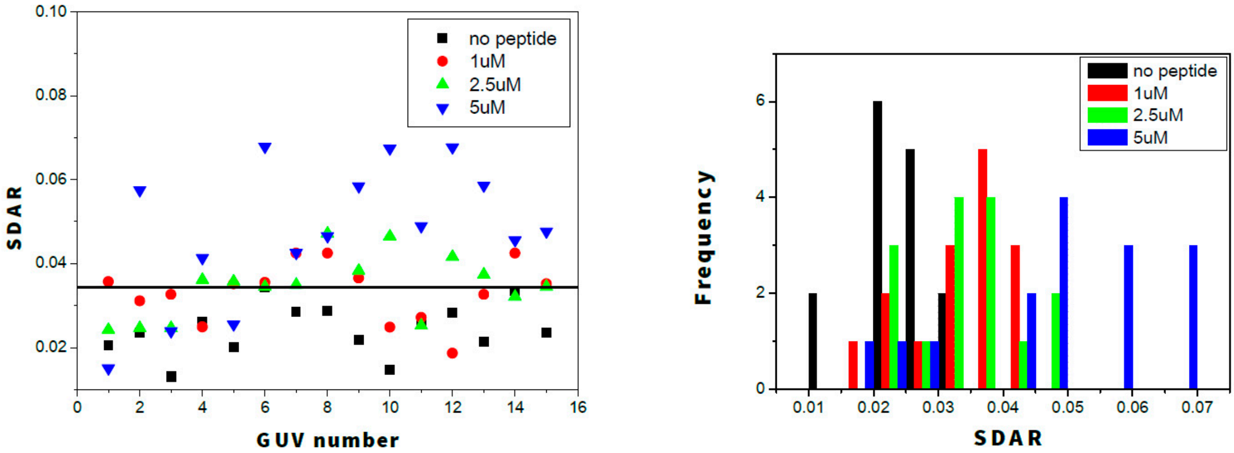

Figure S4: Test for BLM formation

Impedance data were fitted with the following equivalent circuit:

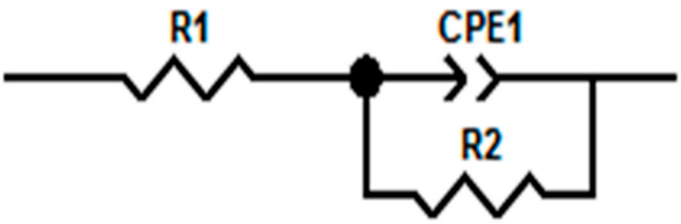

R1: Solution resistance
R2: Hole or BLM resistance
CPE1: Constant Phase Element. The α exponents obtained from the fitting were in the range 0.8-1, thus indicting a capacitive behavior.

A representative fit to the data is shown in the next figure (Nyquist plot) using two different scales:

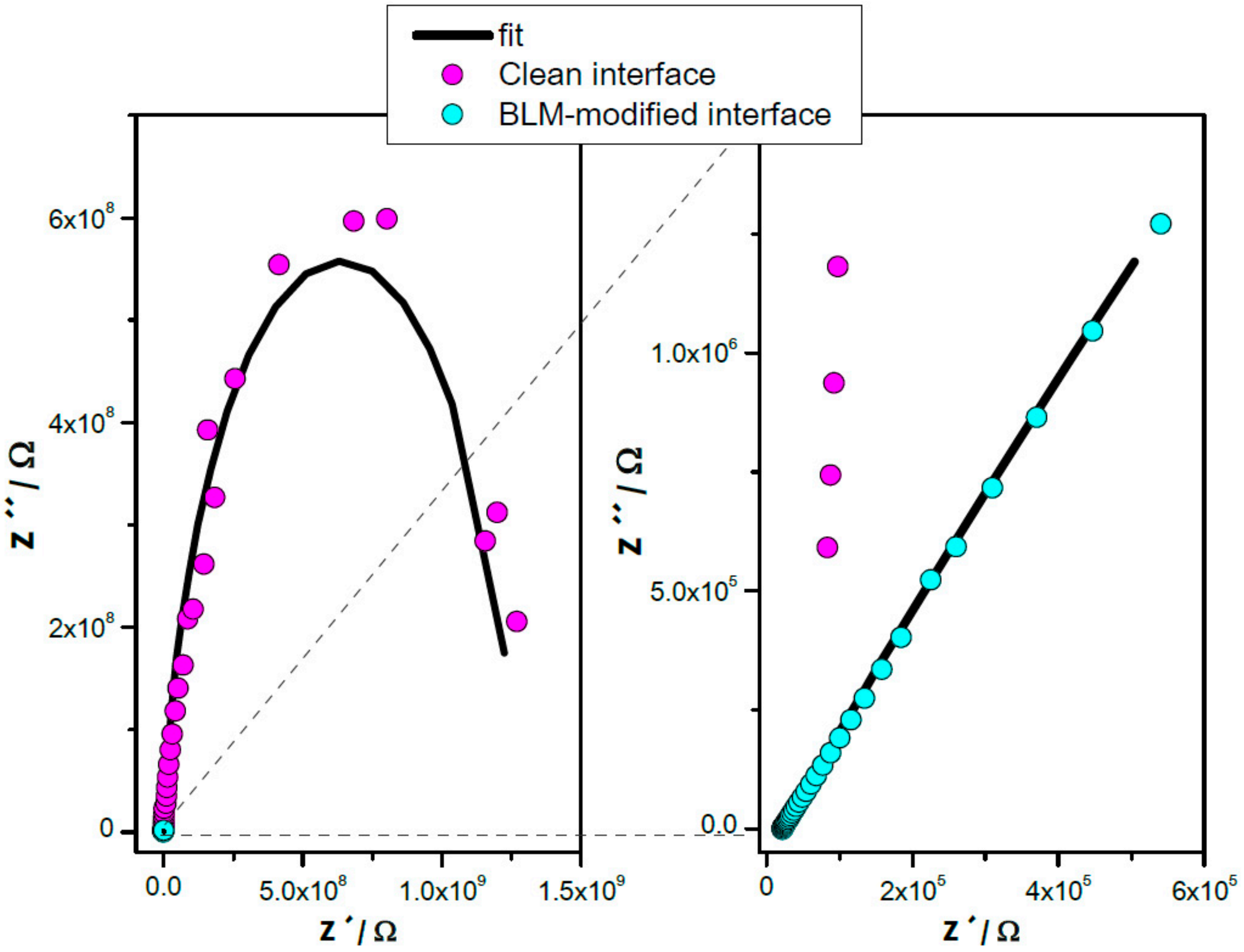

This fitting yielded similar values for R1 (about $1\text{-}5\times10^4$ ∧) and the following parameters:

	CPE / μF cm^{-2}	R2 (Ω)
Clean interface (before BLM formation)	$(3.60\pm0.04)\times10^4$	$(5\pm3)\times10^7$

BLM-modified interface	0.39±0.02	(1300±7)×10^7

Figure S5: Representative conductivity experiments

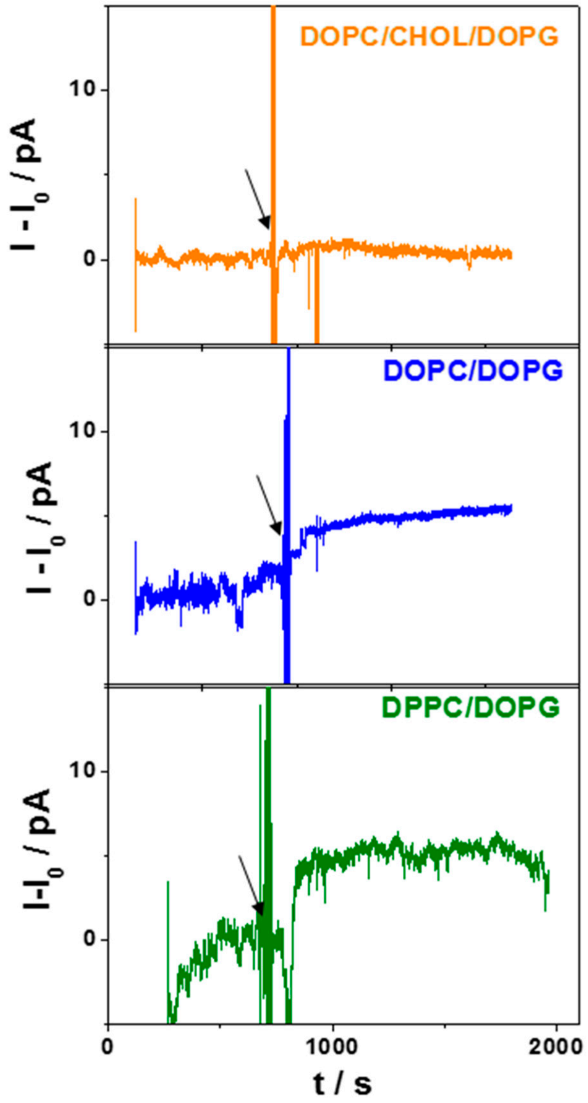

Current as a function of time for BLMs composed of the indicated lipid mixtures. Arrows indicate the time for peptide addition to the cis compartment of the electrochemical cell. A potential of 150 mV (with respect to OCP) is applied during the whole register.

Figure S6: Compression isotherms and calculation of ΔA upon peptide incorporation

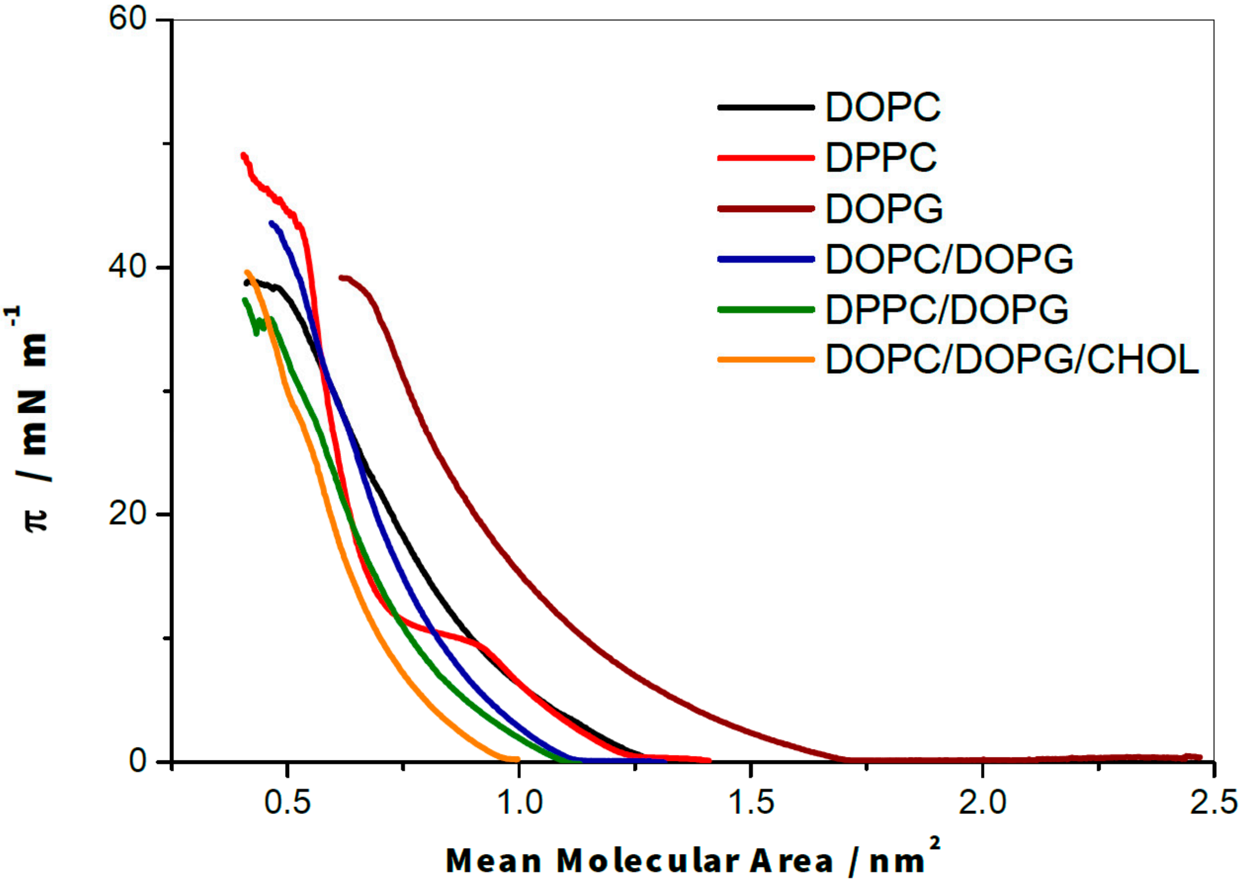

The change in the mean molecular area is calculated considering the monolayer in two regions, a minority one close to the peptide, where the lipid monolayer restructurates, and another region occupied by the majority of the lipids. This region consists of pure lipids that progressively compress as the peptide penetrates due to the change in surface pressure. For these lipids, the area changes from that at π_o to that at π_f. Both areas can be obtained from the compression isotherm of the lipids.

Figure S7: Size distribution of LUVs in the presence of KR_9C

LUV`s size was determined using Dynamic Light Scattering at increasing concentrations of peptide.

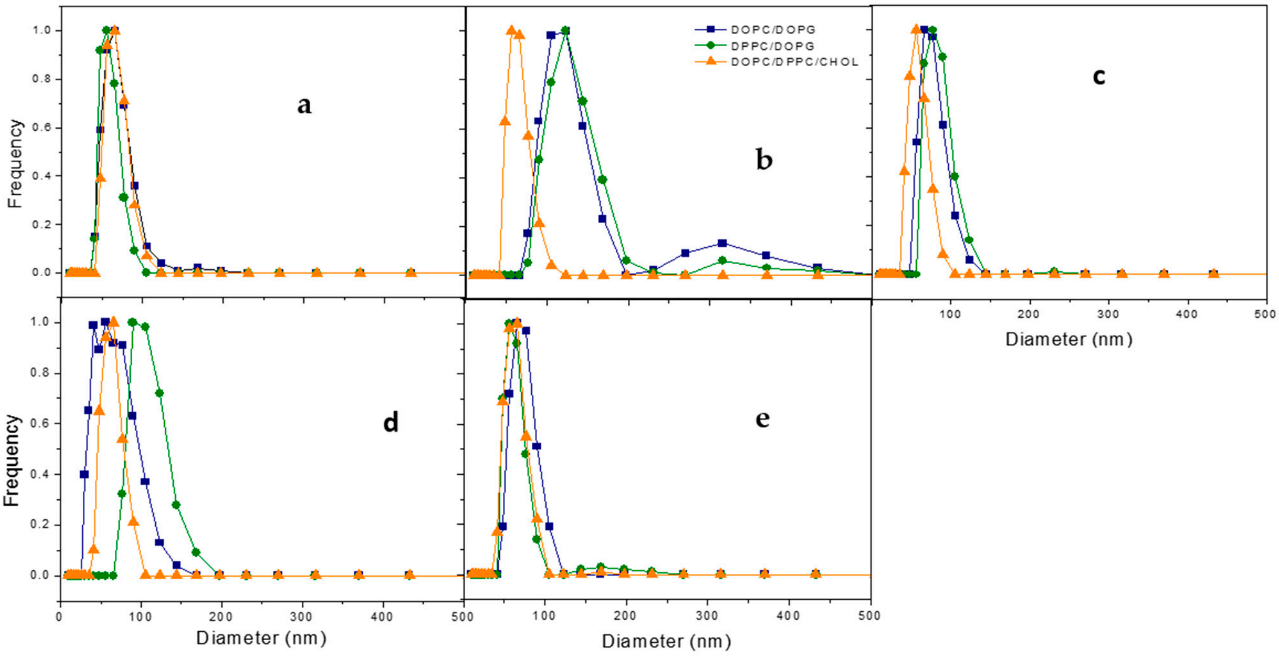

Distribution of vesicle sizes (number weighting) for P/L= 0 (a); $7x10^{-4}$ (b); $3x10^{-2}$(c); $4x10^{-2}$ (d); $1,6x10^{-1}$ (e). Histograms with volume or Intensity weighting also showed a major population around 100 nm, and larger structures appeared.

Figure S8: Kinetic of peptide accumulation at the membrane

The values of *k* were obtained by fitting the plots of Γ_{rim} *vs* time as explained in the manuscript. The figure shows the distribution of the values of *k* for the two analyzed GUV´s compositions.

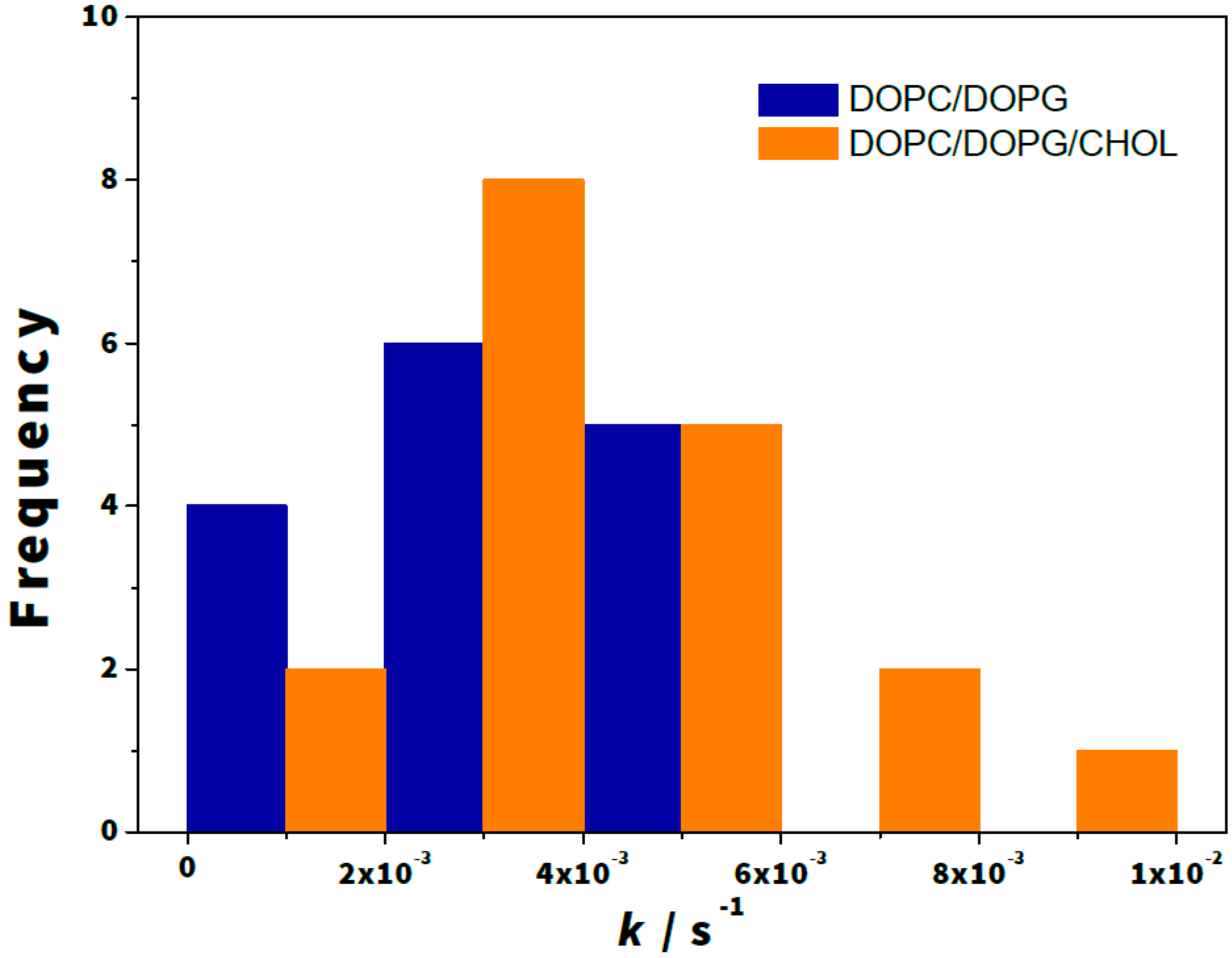

Supplement: Supplementary file 1 [file biomolecules-09-00625-s001.pdf]
